# Ginsenoside Rg1 attenuates adjuvant-induced arthritis in rats via modulation of PPAR-γ/NF-κB signal pathway

**DOI:** 10.18632/oncotarget.19526

**Published:** 2017-07-24

**Authors:** Leiming Zhang, Maojing Zhu, Minmin Li, Yuan Du, Sijin Duan, Yanan Huang, Yongying Lu, Jianqiao Zhang, Tian Wang, Fenghua Fu

**Affiliations:** ^1^ School of Pharmacy, Key Laboratory of Molecular Pharmacology and Drug Evaluation (Yantai University), Ministry of Education, Collaborative Innovation Center of Advanced Drug Delivery System and Biotech Drugs in Universities of Shandong, Yantai University, Yantai, P.R. China

**Keywords:** ginsenoside Rg1, adjuvant-induced arthritis, PPAR-γ, IκBα, NF-κB

## Abstract

Ginsenoside Rg1, the main active compound in *Panax ginseng*, has already been shown to have anti-inflammatory effects. However, the protective effects of Rg1 on rheumatoid arthritis (RA) remain unclear. The aim of the present study was to investigate the effects and mechanisms of Rg1 on adjuvant-induced arthritis (AIA) in rats. AIA rats were given Rg1 at doses of 5, 10, and 20 mg/kg intraperitoneally for 14 days to observe the anti-arthritic effects. The results showed that Rg1 significantly alleviated joint swelling and injuries. Rg1 can also significantly reduce the level of TNF-α and IL-6, increase PPAR-γ protein expression, inhibit IκBα phosphorylation and NF-κB nuclear translocation in the inflammatory joints of AIA rats and RAW264.7 cells stimulated by lipopolysaccharide (LPS). The results indicate that Rg1 has therapeutic effects on AIA rats, and the mechanism might be associated with its anti-inflammatory effects by up-regulating PPAR-γ and subsequent inhibition of NF-κB signal pathway.

## INTRODUCTION

Rheumatoid arthritis (RA) is a chronic destructive disease of the joints. It is characterized by chronic proliferative synovitis, infiltration of inflammatory cells into the synovial tissue of joints, and cartilage destruction. It is here reported that RA affects around 0.5–1% of adults in developed countries with 5–50 per 100,000 new cases each year [1, 2].

Non-steroidal anti-inflammatory drugs (NSAIDs) and glucocorticoids are conventional anti-inflammatory drugs that have been widely used in the treatment of RA. However, in response to recent evidence, NSAIDs have lost their prominent role as first-line treatment because of concerns about their limited effectiveness, inability to alter the long-term course of disease or gastrointestinal or cardiac toxic effects [3, 4]. The adverse effects of long-term glucocorticoid treatment are common and include immunosuppression, osteoporosis, and metabolic disorders [5, 6]. In recent years, biological agents especially tumor necrosis factor (TNF) inhibitors are widely used in the treatment of RA [7]. However, the infection rate and high costs restrict the prescription of biological agents [8, 9].

In the past few years, PPARs have also emerged as key regulators of inflammatory and immune responses, opening up a new area for the development of therapeutic drugs useful to the treatment of chronic inflammatory diseases such as atherosclerosis, obesity-induced insulin resistance and arthritis [10, 11]. Cumulative experimental evidence has proved that natural and synthetic PPAR-γ ligands can inhibit major signaling pathways of inflammation such as NF-κB, which underlies many aspects of the anti-inflammatory effect of PPAR-γ, and reduce the synthesis of cartilage catabolic factors responsible for articular cartilage degradation in arthritis. PPAR-γ is now considered a probable molecular target for the treatment of chronic inflammatory diseases, including arthritis [12].

**Figure 1:**
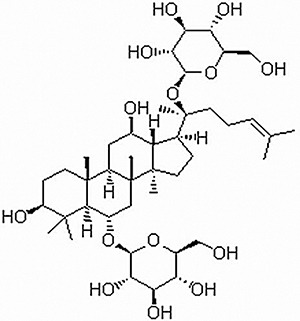
Chemical structure of Rg1

Plants used in traditional medicine currently provide a rich source of candidate drugs for the treatment of chronic inflammatory diseases. Ginseng, the root of *Panax ginseng* C.A. Meyer, is now one of the most extensively used alternative medicines throughout the world [13–15]. The molecular agents responsible for ginseng's actions are ginsenosides [16, 17], which are triterpene saponins that have rigid steroidal skeletons with sugar moieties [18]. Rg1 (Figure 1), the active ingredient of *Panax ginseng* [19], has already been shown to have anti-inflammatory effects. It can inhibit lipopolysaccharide (LPS)-induced sepsis by competitive binding to Toll-like receptor 4 (TLR4), which was found to improve survival in a murine model [20, 21]. It can also ameliorate chronic inflammatory disease such as colitis by inhibiting the binding of LPS to TLR4 on macrophages [22]. Recently, studies reported that Rg1 can suppress inflammation by activating PPAR-γ/HO-1 in rat model of cerebral ischemia-reperfusion injury [23], it can also inhibit the TLR4-NF-κB signaling pathway in the murine neuroglial cell line [24].

However, the protective effects and mechanism of ginsenoside Rg1 on RA are still poorly elucidated. In the present study, we determined whether Rg1 had protective effects on complete Freund's adjuvant (CFA)-induced arthritis in rats. We also carried out an investigation into the mechanisms underlying the therapeutic effect of Rg1 on CFA-induced arthritis, which involve PPAR-γ/NF-κB signaling.

## RESULTS

### Effects of Rg1 on paw swelling in AIA rats

**Figure 2:**
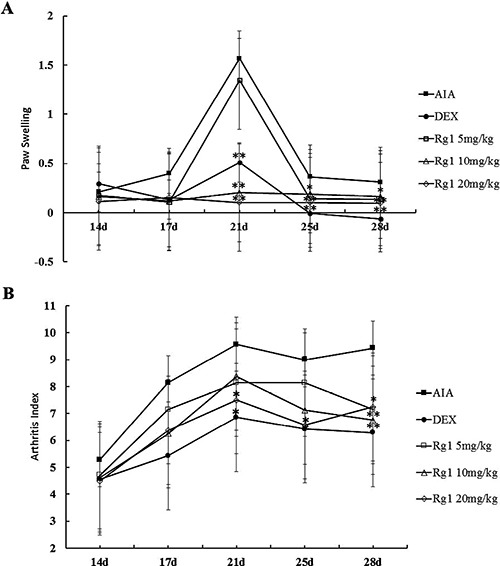
Effects of Rg1 on paw swelling and the arthritis index in AIA rats SD rats were immunized with complete Freund's adjuvant (CFA). The rats were then given Rg1 or DEX from day 15 to day 28. The paw swelling (**A**) and arthritis indexes (**B**) were evaluated on days 14, 17, 21, 25, and 28. Data are expressed as mean ± SD (*n* = 7 for AIA group, 7 for DEX group, 7 for Rg1 5 mg/kg group, 8 for the Rg1 10 mg/kg group, 8 for Rg1 20 mg/kg group). *P < 0.05 and **P < 0.01 versus AIA.

Adjuvant-injected animals exhibited marked secondary arthritis of the left paw from day 14. DEX 2.5 mg/kg notably inhibited paw swelling at days 17, 21, 25, and 28. Rg1 10 mg/kg and 20 mg/kg notably inhibited paw swelling at days 21, 25, and 28. Rg1 5 mg/kg produced notable inhibition of paw swelling at days 28 (Figure 2A).

### Effects of Rg1 on arthritis index in AIA rats

Fully developed arthritis including redness and swollen paws were observed from day 14. Rats in the AIA group showed severe inflammation with marked lesions and deformity. Secondary lesions such as inflammation of the left paw and nodule formation were also observed. DEX 2.5 mg/kg produced notable inhibition of the arthritis index at days 21, 25, and 28. Rg1 20 mg/kg produced notable inhibition of the arthritis index at days 25 and 28. Rg1 10 mg/kg produced notable inhibition at day 28 (Figure 2B).

### Effects of Rg1 on radiography of the joints of AIA rats

**Figure 3:**
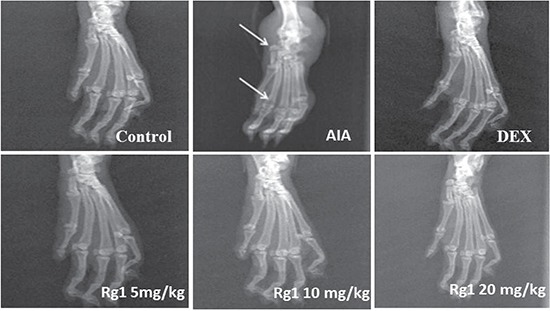
Effects of Rg1 on radiography of the joints of AIA rats SD rats were immunized with complete Freund's adjuvant (CFA). The rats were then given Rg1 or DEX from day 15 to day 28. On day 28, rats were anesthetized with chloral hydrate, then placed on a radiographic box at a distance of 90 cm from the X-ray source. Radiographic analysis were performed by X-ray machine.

Compared with the control group rats, bone matrix resorption, osteophyte formation and bone erosion can be observed in the joints of AIA group rats. After Rg1 treatment at doses of 10 and 20 mg/kg, bone erosion and the degree of arthritis were significantly reduced (Figure 3).

### Effects of Rg1 on histopathology of the joints of AIA rats

**Figure 4:**
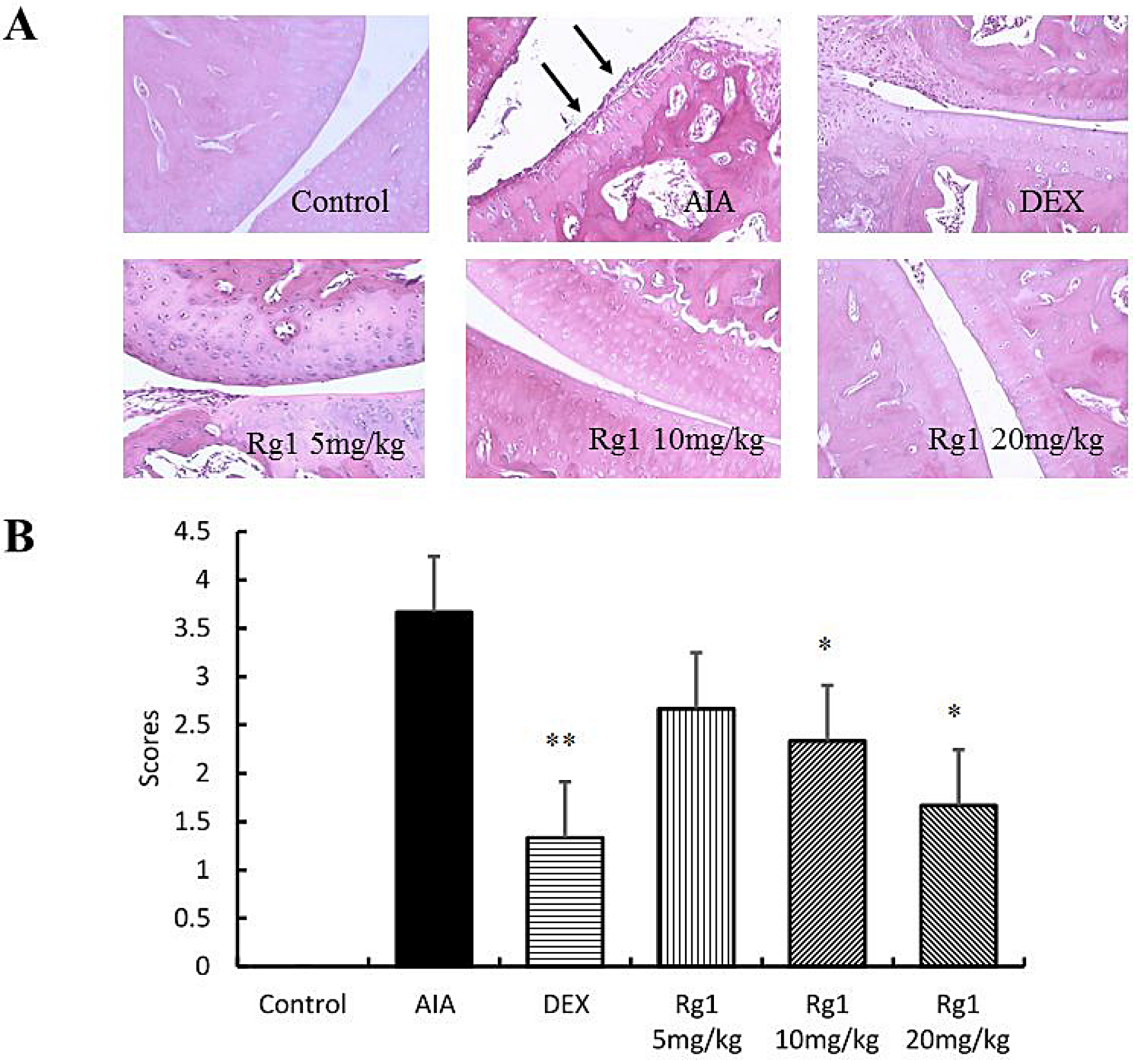
Effects of Rg1 on joint histopathology of AIA rats (**A**) Representative histopathologies of ankle joints stained with H-E (original magnification, 200×). (**B**) Histologic scores of the joint were evaluated. Data are expressed as mean ± SD from 3 animals for each group. *P < 0.05 and **P < 0.01 versus AIA; ^ΔΔ^*P* < 0.01 versus control.

The ankle joints of rats in the control group showed intact cartilage, bone, and synovium. Histologic evaluation of the ankle joints in AIA group revealed adipose marrow mesenchymal cells with embedded inflammatory cells, synovium with multiple giant cells, massive periosteal proliferation, periarticular inflammation, and pannus formation. In the rats that received Rg1 at doses of 10 mg/kg and 20 mg/kg, the degree of arthritis was significantly reduced. This indicated a marked decrease of synovial inflammatory cell infiltrate, synovial lining hyperplasia, and bone destruction (Figure 4).

### Effects of Rg1 on TNF-α and IL-6 levels *in vivo* and *in vitro*

**Figure 5:**
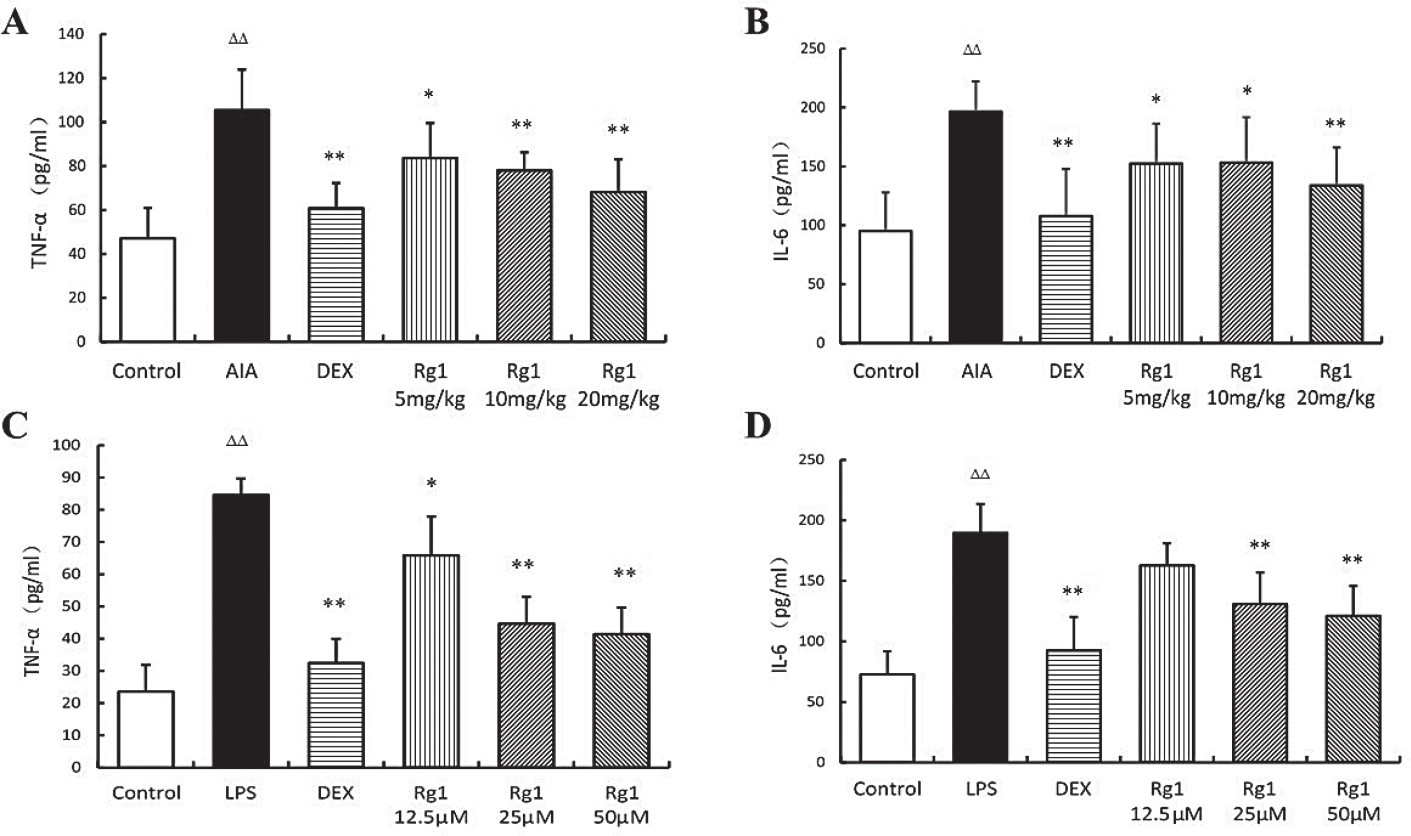
Effects of Rg1 on TNF-α and IL-6 levels *in vivo* and *in vitro* (**A**–**B**) Effects of Rg1 on TNF-α and IL-6 levels in AIA rats (*n* =7 for each group); (**C**–**D**) Effects of Rg1 on TNF-α and IL-6 levels in RAW264.7 cells treated with LPS (*n* = 5 for each group). Data are expressed as mean ± SD. *P < 0.05 and **P < 0.01 versus AIA or LPS; ^ΔΔ^*P* < 0.01 versus control.

The concentration of TNF-α and IL-6 was significantly higher in AIA rats than in control rats (*P* < 0.01). However, Rg1 appeared to decreased the TNF-α and IL-6 levels significantly (*P* < 0.05 or *P* < 0.01). The same results were observed in RAW264.7 cells treated with LPS (Figure 5).

### Effects of Rg1 on p-IκBα, p-p65, and PPAR-γ protein levels *in vivo* and *in vitro*

**Figure 6:**
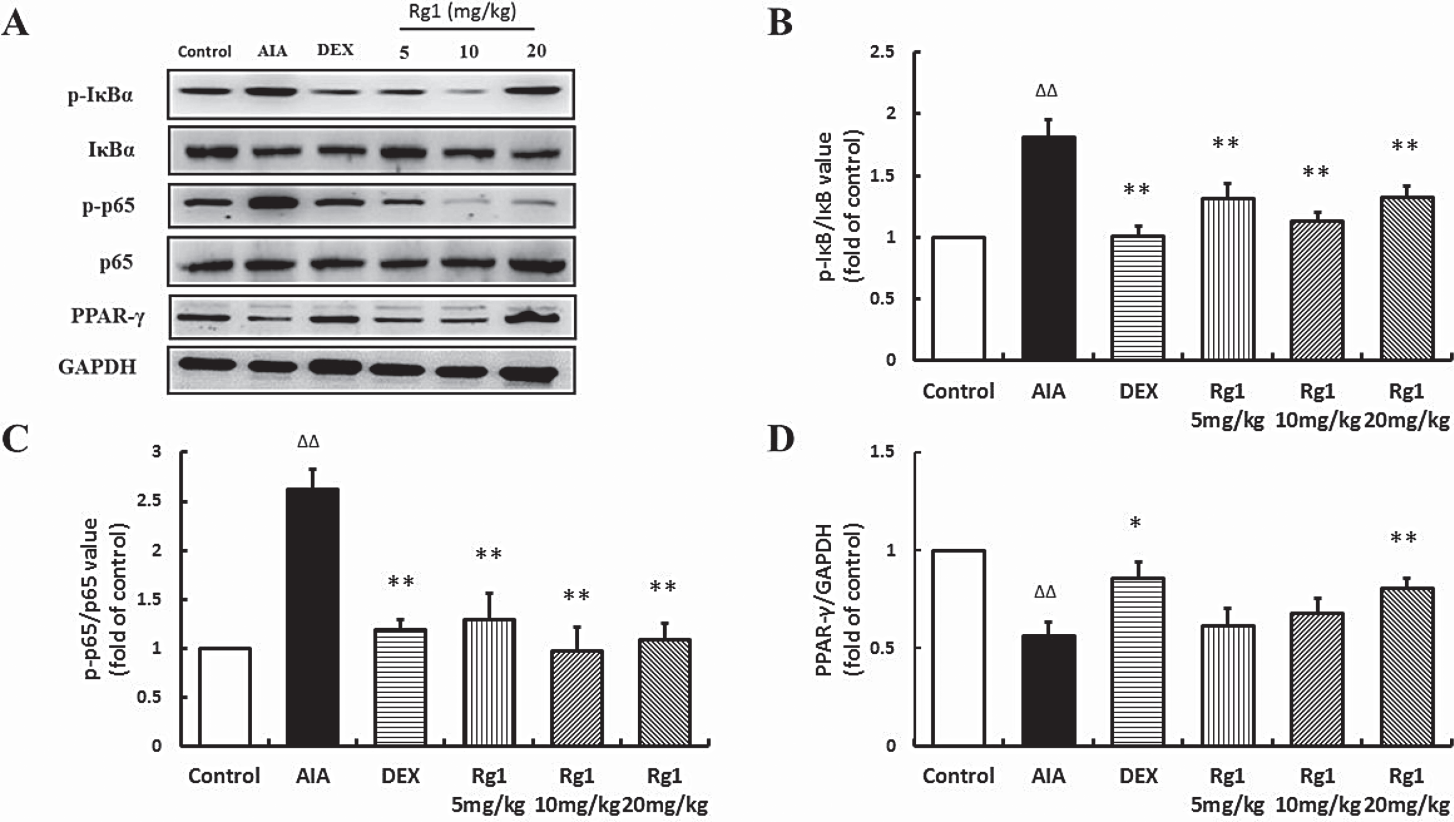
Effects of Rg1 on p-IκBα, p-p65, and PPAR-γ protein expression in AIA rats (**A**) Western blot bands; (**B**) p-IκBα expression; (**C**) p-p65 expression; (**D**) PPAR-γ protein expression. Data are expressed as mean ± SD, *n* = 3 for each group. *P < 0.05 and **P < 0.01 versus AIA; ^ΔΔ^*P* < 0.01 versus control.

**Figure 7:**
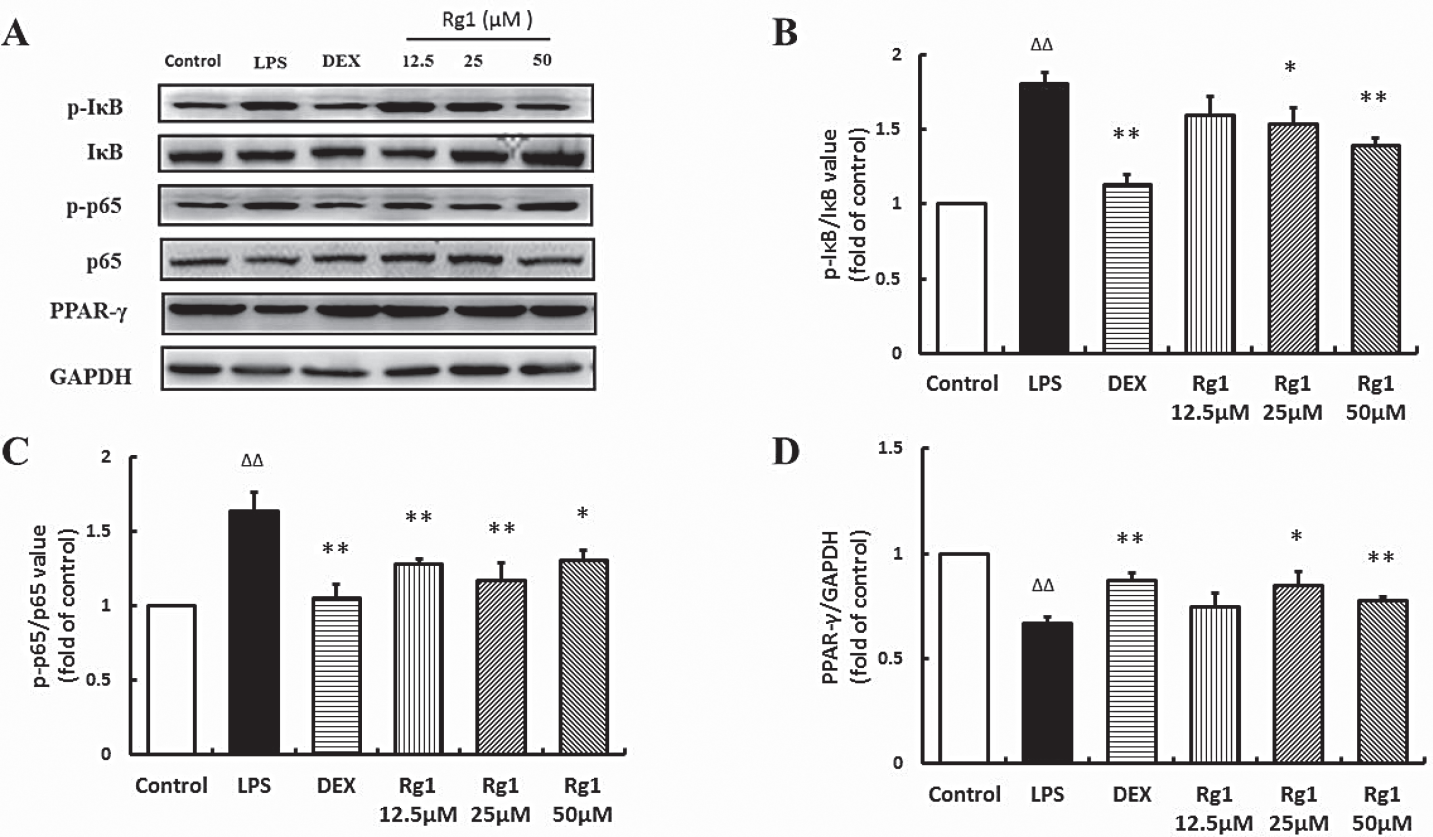
Effects of Rg1 on p-IκBα, p-p65, and PPAR-γ protein expression in RAW264.7 cells treated with LPS (**A**) Western blot bands; (**B**) p-IκBα expression; (**C**) p-p65 expression; (**D**) PPAR-γ protein expression. Data are expressed as mean ± SD, *n* = 3 for each group. *P < 0.05 and **P < 0.01 versus LPS; ^ΔΔ^*P* < 0.01 versus control.

In the inflamed joints of AIA rats and RAW264.7 cells stimulated by LPS, the level of p-IκBα and p-p65 were significantly higher than in controls and PPAR-γ levels were significantly lower. Treatment with Rg1 *in vivo* and *in vitro* inhibited IκBα phosphorylation, reduced NF-κB nuclear translocation and upregulated PPAR-γ expression (Figure 6, Figure 7).

### Effects of Rg1 on index of thymus and spleen in AIA rats

**Figure 8:**
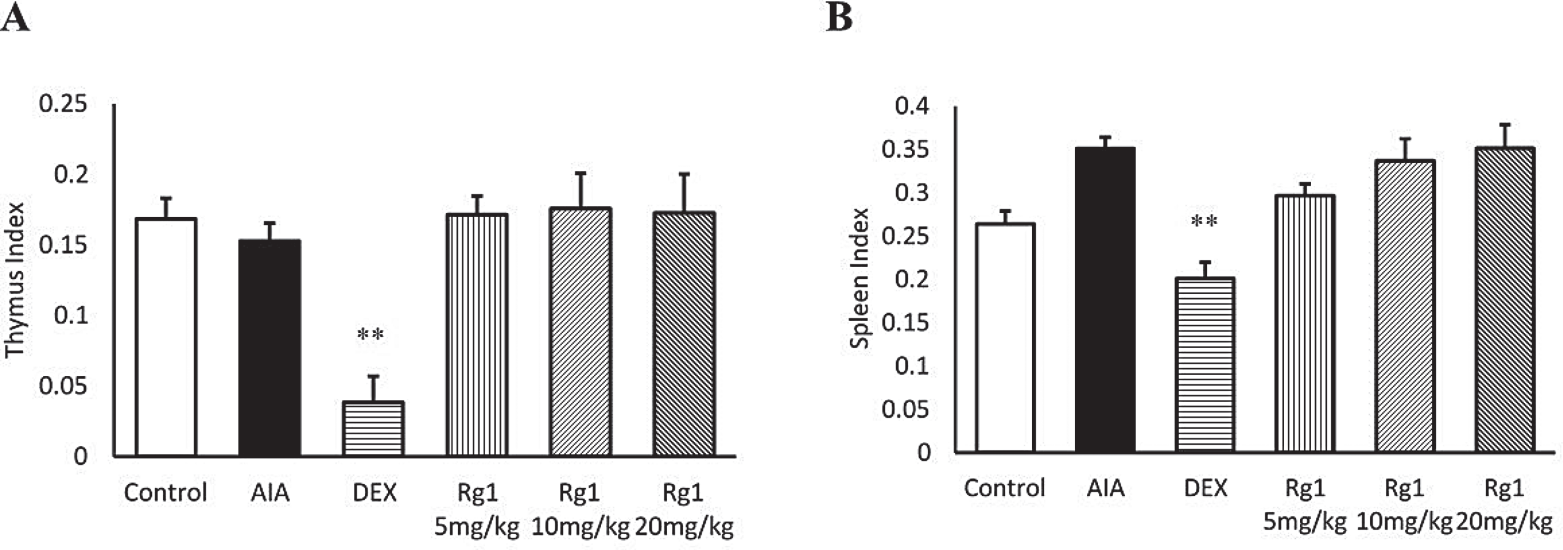
Rg1 and DEX on the thymus and spleen index of AIA rats The index of thymus (**A**) and spleen (**B**) were expressed as the ratio (mg/g) of thymus and spleen wet weight versus body weight, respectively. Data are expressed as mean ± SD from 7 animals for each group. **P < 0.01 versus AIA.

The index of thymus and spleen in DEX administration rats was markedly lower than in AIA rats (*P* < 0.01). However, rats given Rg1 showed no significant differences from AIA rats (Figure 8).

## DISCUSSION

RA is an autoimmune and chronic inflammatory disease; it is pathologically characterized by synovial hyperplasia, inflammation, and angiogenesis in synovial membrane that leads to pannus formation followed by severe cartilage and bone destruction [25].

Adjuvant-induced arthritis is a commonly used animal model for study of the pathogenesis of human RA and also for finding novel drugs for RA management [26]. Complete Freund’s-adjuvant-induced arthritis is an important animal model of screening anti-inflammatory drugs due to its histological and immunological similarity to RA in human patients [27]. In the present study, adjuvant-injected animals exhibited marked secondary arthritis. Histologic evaluation of the ankle joints revealed massive periosteal proliferation, periarticular inflammation, and pannus formation. Rg1 significantly reduced paw swelling, synovial inflammatory cell infiltration, synovial lining hyperplasia, and bone destruction, which indicates Rg1 has good anti-arthritic effects.

Inflammatory cytokines, such as TNF-α, IL-1β, and IL-6, have proangiogenic activity that promotes synovial inflammation by increasing the expression of vascular growth factors, endothelial mitogenesis, and induction of matrix metalloproteinases [28]. They also supply nutrients and oxygen to increase inflammatory cell mass and facilitate the infiltration of inflammatory cells into the synovial tissues to enhance the inflammatory response [29]. In the present study, both DEX and Rg1 significantly inhibited TNF-α and IL-6 concentration in serum of AIA rats and inflammatory cytokine secretion by RAW 264.7 macrophage cells *in vitro*. However, glucocorticoids, as conventional anti-inflammatory drugs, have adverse effects of long-term glucocorticoid treatment including immunosuppression. In the present study, the index of thymus and spleen in DEX administration rats was markedly lower than in AIA rats. But Rg1 showed no significant immunosuppressive effects compared with DEX. The results indicate that Rg1 has good anti-inflammatory effects but no immunosuppressive effects. Currently, the precise mechanisms by which Rg1 exerts its anti-inflammatory effects on AIA rats remain unclear.

Many signal transduction pathways are activated in RA synovial tissue, and the NF-κB pathway is one of the most important to the pathogenesis of RA [30]. The NF-κB family contains 5 related transcription factors. These normally bind to IκB in the cytoplasm. Stress, inflammatory cytokines, and microbial products result in IκB phosphorylation and degradation, allowing NF-κB to translocate to the nucleus and regulate gene transcription. NF-κB plays crucial roles in the regulation of inflammation and immune responses, and inappropriate NF-κB activity has been linked to many autoimmune and inflammatory diseases, including RA [31]. NF-κB is induced by many stimuli, including TNF-α and IL-6, forming a positive regulatory cycle that may amplify and maintain RA disease process by producing more inflammatory cytokines. NF-κB and the enzymes involved in its activation may be suitable targets for anti-arthritic treatment [32].

PPAR-γ is a transcription factor that acts as an influential pleiotropic regulator of anti-inflammation, antioxidant, and phagocyte mediated cleanup processes. In various experimental models of arthritis, PPAR-γ activation has been shown to exhibit an anti-inflammatory effect and reduce the severity of the disease [33, 34]. Apart from its direct genomic effect, PPAR-γ has been found to interact negatively with other transcription factors such as NF-κB, which underlies many aspects of the anti-inflammatory effect of PPAR-γ [35, 36]. Many reports have documented that activated PPAR-γ can inhibited NF-κB signaling pathways to protect against RA [37, 38]. These findings show that treatment with Rg1 *in vivo* and *in vitro* can upregulate PPAR-γ expression, inhibit IκBα phosphorylation, and reduce NF-κB nuclear translocation, which has suggested that the anti-inflammatory effects of Rg1 in AIA rats are associated with its ability to modulate PPAR-γ and NF-κB transcription factors.

In summary, Rg1 has therapeutic effects on adjuvant-induced arthritis in rats. The mechanism might be associated with its anti-inflammatory effects via upregulation of PPAR-γ expression and subsequent inhibition of NF-κB signal pathway.

## MATERIALS AND METHODS

### Animals

Male Sprague–Dawley (SD) rats (weight, 150–170 g) were purchased from Beijing HFK Bioscience Co., Ltd. (Beijing, China). All animals were acclimated for at least 1 week at temperature of 24 ± 1°C and humidity of 55 ± 5%. The animals were maintained with free access to standard diet and tap water. The experimental procedures were approved by the Animal Ethics Committee of Yantai University.

### Reagents and drugs

Ginsenoside Rg1 (Formula: C_42_H_72_O_14_, Figure 1) was purchased from Nanjing Guangrun Biotechnology Co., Ltd. (Nanjing, China) with a purity of ≥ 99% as determined by HPLC. The dexamethasone sodium phosphate injections were purchased from the Cisen Pharmaceutical Co. Ltd. (China). Complete Freund's adjuvant (CFA) (containing 10 mg/mL of dry, heat-killed *Mycobacterium tuberculosis*) was purchased from Chondrex, Inc. (U.S.). Lipopolysaccharides (LPS) were purchased from Sigma-Aldrich Corporation (U.S.). TNF-α and IL-6 ELISA kits were purchased from Nanjing Jiancheng Bioengineering Institute (Nanjing, China). PPAR-γ, IκBα, p-IκBα and NF-κB (p65 and p-p65) antibodies were obtained from Santa Cruz Biotechnology, Inc. (U.S.). The other chemicals were of analytical grade.

### Induction of adjuvant arthritis

Arthritis was induced by a single intradermal injection of 0.1 ml of CFA (10 mg/ml) emulsion into the right hind metatarsal footpad of SD rats. The control group rats were injected with saline. The day of the first immunization was defined as day 0 [39].

### Drug administration

Rats were divided into 6 groups, including Control, AIA, dexamethasone (DEX, 2.5 mg/kg), Rg1 5 mg/kg, Rg1 10 mg/kg, and Rg1 20 mg/kg (Rg1 doses were selected according to the previous study [20–22]). After the onset of arthritis, rats were given Rg1 and DEX intraperitoneally once daily for 14 days.

### Evaluation of arthritis

To evaluate the severity of arthritis, paw swelling and arthritis indexes were evaluated on days 14, 17, 21, 25, and 28, by 2 observers blind to treatment. Paw swelling was measured with a plethysmometer (YLS-7A, Shandong Academy of Medical Sciences, China). The results were expressed according to the increase in paw volume (mL) calculated by subtracting the basal volume. Inflammation of the 4 paws was graded from 0 to 4 according to previous studies [40]: 0, paws with no swelling or focal redness; 1, swelling of the finger joints; 2, mild swelling of the ankle or wrist joints; 3, severe inflammation of the entire paw; 4, paws with deformity or ankylosis. Each paw was scored separately and the cumulative scores of all 4 paws of each rat served as the polyarthritis index with a maximum value of 16 per rat.

### Radiography

On day 28, rats were anesthetized with chloral hydrate, then placed on a radiographic box at a distance of 90 cm from the X-ray source. Radiographic analysis were performed by X-ray machine.

### Histological examination

After chloral hydrate anesthesia on Day 28, animals were sacrificed by cervical dislocation. Legs were severed under the knee, flayed, and fixed in 10% neutral-buffered formalin for 48 h at room temperature, then decalcified in 5% formic acid and embedded in paraffin. The sections (5 μm) were stained with hematoxylin and eosin (H-E) and analyzed qualitatively by 2 independent observers in a blind fashion.

The severity of arthritis in the joint was graded from 0 to 4 according to the intensity of lining layer hyperplasia, mononuclear cell infiltration, and pannus formation, as described previously: 0 = normal ankle joint, 1 = normal synovium with occasional mononuclear cells, 2 = definite arthritis with a few layers of flat to rounded synovial lining cells and scattered mononuclear cells and dense infiltration of mononuclear cells, 3 = clear hyperplasia of the synovium with 3 or more layers of loosely arranged lining cells and dense infiltration with mononuclear cells, 4 = severe synovitis with pannus and erosion of articular cartilage and subchondral bone [41].

### Cytokine determination

Measurement of TNF-α and IL-6 in blood of AIA rats: All rats were sacrificed on day 28 after chloral hydrate anesthesia, and blood was collected from the abdominal aorta for determination of TNF-α and IL-6 levels using ELISA kits according to the manufacturer's' instructions.

RAW 264.7 cells culture and cytokine determination: The mouse monocyte-macrophage cell line RAW 264.7 was obtained from the cell library in the Shanghai Institute of Cell Biology, Chinese Academy of Science (Shanghai, China). The cells were cultured in RPMI 1640 supplemented with 10% FBS, 100 U/ml penicillin, and 0.1 mg/ml streptomycin. Cells were kept at 37°C with 5% CO_2_ in a fully humidified atmosphere. The medium was routinely changed every day. Cell viability assay: Cells (concentration: 1 × 10^6^ cells /ml) were treated with Rg1 at concentrations of 1–100 μM. The results showed Rg1 did not exhibit cytotoxicity (data not shown). So this concentration range was used in the following experiments. Cells were treated with LPS (1 μg/ml) with or without drugs (Rg1, 12.5 μM, 25 μM, and 50 μM) for 24 h. Then 100 μl of the culture supernatant was removed to determine the level of TNF-α and IL-6 using ELISA assay kits according to the manufacturer's instructions.

### Western blot analysis

Tissues of inflamed joints were separated and homogenized on ice in cold lysis buffer (Beyotime, China) plus 1:100 volume of phenylmethyl sulfonylfluoride (PMSF). RAW 264.7 cells were treated with LPS (1 μg/ml) and Rg1 (12.5 μM, 25 μM, and 50 μM) for 24 h and then washed with cold PBS and lysed in ice-cold lysis buffer (Beyotime, China) plus 1:100 volume of PMSF. The supernatant was aliquoted and protein concentrations were determined using a bicinchoninic acid (BCA) protein assay kit (Beyotime, China). Proteins were segregated by electro-blotted and SDS-PAGE into a membrane of nitrocellulose. The blots were probed with antibodies against PPAR-γ, IκBα, p-IκBα, NF-κB p65, p-p65 (all antibodies were diluted by 1:1000) at 4°C overnight and later incubated with horseradish peroxidase-conjugated IgG secondary antibody (Beyotime, China; 1:5000 dilution) for 1 h. The expression of every protein was detected by the detection system of ECL (ChemiDoc^™^XRS, Bio-Rad, Shanghai, China). The respective bands were quantitated and images were collected utilizing Quantity One Software (Bio-Rad). The fold increase over control was used to express the results [42].

### Index of thymus and spleen

On day 28, the animals were sacrificed via chloral hydrate anesthesia. The thymus and spleen were promptly removed and weighed. The index of thymus and spleen were expressed as the ratio (mg/g) of thymus and spleen wet weight versus body weight, respectively.

### Statistical analysis

The data are expressed as means ± SD. Data were analyzed using one-way ANOVA with Bonferroni post-hoc testing for multiple *t*-tests. *P* < 0.05 was considered indicative of statistically significant differences among groups.

## Abbreviations

RA: rheumatoid arthritis
AIA: adjuvant-induced arthritis
DEX: dexamethasone
TNF-α: tumor necrosis factor-α
IL-6: interleukin-6
PPAR-γ: peroxisome proliferator-activated receptor γ
NF-κB: nuclear factor-kappa B
IκB: inhibitor kappa b
LPS: lipopolysaccharide
NSAIDs: non-steroidal anti-inflammatory drugs
TLR4: toll like receptor 4
MAPK: mitogen-activated protein kinase
CFA: complete Freund's adjuvant.

## References

[1] Scott DL, Wolfe F, Huizinga TW (2010). Rheumatoid arthritis. Lancet.

[2] Fu Y, Zhou H, Wang M, Cen J, Wei Q (2014). Immune regulation and anti-inflammatory effects of isogarcinol extracted from Garcinia mangostana L. against collagen-induced arthritis. J Agric Food Chem.

[3] Scott PA, Kingsley GH, Smith CM, Choy EH, Scott DL (2007). Non-steroidal anti-inflammatory drugs and myocardial infarctions: comparative systematic review of evidence from observational studies and randomised controlled trials. Ann Rheum Dis.

[4] Schaffer D, Florin T, Eagle C, Marschner I, Singh G, Grobler M, Fenn C, Schou M, Curnow KM (2006). Risk of serious NSAID-related gastrointestinal events during long-term exposure: a systematic review. Med J Aust.

[5] Baschant U, Culemann S, Tuckermann J (2013). Molecular determinants of glucocorticoid actions in inflammatory joint diseases. Mol Cell Endocrinol.

[6] Baschant U, Lane NE, Tuckermann J (2012). The multiple facets of glucocorticoid action in rheumatoid arthritis. Nat Rev Rheumatol.

[7] Feldmann M, Maini SR (2008). Role of cytokines in rheumatoid arthritis: an education in pathophysiology and therapeutics. Immunol Rev.

[8] Atzeni F, Batticciotto A, Masala IF, Talotta R, Benucci M, Sarzi-Puttini P (2016). Infections and Biological Therapy in Patients with Rheumatic Diseases. Isr Med Assoc J.

[9] Cohen MD, Keystone E (2015). Rituximab for Rheumatoid Arthritis. Rheumatol Ther.

[10] Daynes RA, Jones DC (2002). Emerging roles of PPARs in inflammation and immunity. Nat Rev Immunol.

[11] Kostadinova R, Wahli W, Michalik L (2005). PPARs in diseases: control mechanisms of inflammation. Curr Med Chem.

[12] Giaginis C, Giagini A, Theocharis S (2009). Peroxisome proliferator-activated receptor-gamma (PPAR-gamma) ligands as potential therapeutic agents to treat arthritis. Pharmacol Res.

[13] Seo JY, Ju SH, Oh J, Lee SK, Kim JS (2016). Neuroprotective and Cognition-Enhancing Effects of Compound K Isolated from Red Ginseng. J Agric Food Chem.

[14] Lee YY, Park JS, Lee EJ, Lee SY, Kim DH, Kang JL, Kim HS (2015). Anti-inflammatory mechanism of ginseng saponin metabolite Rh3 in lipopolysaccharide-stimulated microglia: critical role of 5′-adenosine monophosphate-activated protein kinase signaling pathway. J Agric Food Chem.

[15] Lee SY, Jeong JJ, SH Le TH Eun, Nguyen MD, Park JH, Kim DH (2015). Ocotillol, a Majonoside R2 Metabolite, Ameliorates 2,4,6-Trinitrobenzenesulfonic Acid-Induced Colitis in Mice by Restoring the Balance of Th17/Treg Cells. J Agric Food Chem.

[16] Jia L, Wang WY, Zhou LM, Mo FF, Li M (2010). [Antimotion sickness effects of ginsenosides combined with dexamethasone in rats] [Article in Chinese]. Zhong Xi Yi Jie He Xue Bao.

[17] Kuang HY, Shao H, Hou LH, Wu XK (2008). [Effects of ginseng total saponins on nerve growth factor expression in rat with estradiol valerate-induced polycystic ovaries] [Article in Chinese]. Zhong Xi Yi Jie He Xue Bao.

[18] Ling C, Li Y, Zhu X, Zhang C, Li M (2005). Ginsenosides may reverse the dexamethasone-induced down-regulation of glucocorticoid receptor. Gen Comp Endocrinol.

[19] Sengupta S, Toh SA, Sellers LA, Skepper JN, Koolwijk P, Leung HW, Yeung HW, Wong RN, Sasisekharan R, Fan TP (2004). Modulating angiogenesis: the yin and the yang in ginseng. Circulation.

[20] Zou Y, Tao T, Tian Y, Zhu J, Cao L, Deng X, Li J (2013). Ginsenoside Rg1 improves survival in a murine model of polymicrobial sepsis by suppressing the inflammatory response and apoptosis of lymphocytes. J Surg Res.

[21] Su F, Xue Y, Wang Y, Zhang L, Chen W, Hu S (2015). Protective effect of ginsenosides Rg1 and Re on lipopolysaccharide-induced sepsis by competitive binding to Toll-like receptor 4. Antimicrob Agents Chemother.

[22] Lee SY, Jeong JJ, Eun SH, Kim DH (2015). Anti-inflammatory effects of ginsenoside Rg1 and its metabolites ginsenoside Rh1 and 20(S)-protopanaxatriol in mice with TNBS-induced colitis. Eur J Pharmacol.

[23] Yang Y, Li X, Zhang L, Liu L, Jing G, Cai H (2015). Ginsenoside Rg1 suppressed inflammation and neuron apoptosis by activating PPARγ/HO-1 in hippocampus in rat model of cerebral ischemia-reperfusion injury. Int J Clin Exp Pathol.

[24] Zhao BS, Liu Y, Gao XY, Zhai HQ, Guo JY, Wang XY (2014). Effects of ginsenoside Rg1 on the expression of toll-like receptor 3, 4 and their signalling transduction factors in the NG108-15 murine neuroglial cell line. Molecules.

[25] McInnes IB, Schett G (2011). The pathogenesis of rheumatoid arthritis. N Engl J Med.

[26] Hegen M, Keith JC, Collins M, Nickerson-Nutter CL (2008). Utility of animal models for identification of potential therapeutics for rheumatoid arthritis. Ann Rheum Dis.

[27] Gómez-SanMiguel AB, Gomez-Moreira C, Nieto-Bona MP, Fernández-Galaz C, MÁ Villanúa, Martín AI, López-Calderón A (2016). Formoterol decreases muscle wasting as well as inflammation in the rat model of rheumatoid arthritis. Am J Physiol Endocrinol Metab.

[28] Pan R, Dai Y, Gao X, Xia Y (2009). Scopolin isolated from Erycibe obtusifolia Benth stems suppresses adjuvant-induced rat arthritis by inhibiting inflammation and angiogenesis. Int Immunopharmacol.

[29] Maruotti N, Cantatore FP, Crivellato E, Vacca A, Ribatti D (2006). Angiogenesis in rheumatoid arthritis. Histol Histopathol.

[30] Tak PP, Firestein GS (2001). NF-kappaB: a key role in inflammatory diseases. J Clin Invest.

[31] Abu-Amer Y, Darwech I, Otero J (2008). Role of the NF-kappaB axis in immune modulation of osteoclasts and bone loss. Autoimmunity.

[32] Rasheed Z, Haqqi TM (2008). Update on Targets of Biologic Therapies for Rheumatoid Arthritis. Curr Rheumatol Rev.

[33] Koufany M, Jouzeau JY, Moulin D (2014). Fenofibrate vs pioglitazone: Comparative study of the anti-arthritic potencies of PPAR-alpha and PPAR-gamma agonists in rat adjuvant-induced arthritis. Biomed Mater Eng.

[34] Marder W, Khalatbari S, Myles JD, Hench R, Lustig S, Yalavarthi S, Parameswaran A, Brook RD, Kaplan MJ (2013). The peroxisome proliferator activated receptor-γ pioglitazone improves vascular function and decreases disease activity in patients with rheumatoid arthritis. J Am Heart Assoc.

[35] Huang T, Gao D, Hei Y, Zhang X, Chen X, Fei Z (2016). D-allose protects the blood brain barrier through PPARγ-mediated anti-inflammatory pathway in the mice model of ischemia reperfusion injury. Brain Res.

[36] Kim JS, Lee YH, Chang YU, Yi HK (2017). PPARγ regulates inflammatory reaction by inhibiting the MAPK/NF-κB pathway in C2C12 skeletal muscle cells. J Physiol Biochem.

[37] Shiojiri T, Wada K, Nakajima A, Katayama K, Shibuya A, Kudo C, Kadowaki T, Mayumi T, Yura Y, Kamisaki Y (2002). PPAR gamma ligands inhibit nitrotyrosine formation and inflammatory mediator expressions in adjuvant-induced rheumatoid arthritis mice. Eur J Pharmacol.

[38] Ji JD, Cheon H, Jun JB, Choi SJ, Kim YR, Lee YH, Kim TH, Chae IJ, Song GG, Yoo DH, Kim SY, Sohn J (2001). Effects of peroxisome proliferator-activated receptor-gamma (PPAR-gamma) on the expression of inflammatory cytokines and apoptosis induction in rheumatoid synovial fibroblasts and monocytes. J Autoimmun.

[39] Perera PK, Peng C, Xue L, Li Y, Han C (2011). *Ex vivo* and *in vivo* effect of Chinese herbal pill Yi Shen Juan Bi (YJB) on experimental arthritis. J Ethnopharmacol.

[40] Chang Y, Wu Y, Wang D, Wei W, Qin Q, Xie G, Zhang L, Yan S, Chen J, Wang Q, Wu H, Xiao F, Sun W (2011). Therapeutic effects of TACI-Ig on rats with adjuvant-induced arthritis via attenuating inflammatory responses. Rheumatology (Oxford).

[41] Wu H, Chen J, Song S, Yuan P, Liu L, Zhang Y, Zhou A, Chang Y, Zhang L, Wei W (2016). β2-adrenoceptor signaling reduction in dendritic cells is involved in the inflammatory response in adjuvant-induced arthritic rats. Sci Rep.

[42] Li M, Lu C, Zhang L, Zhang J, Du Y, Duan S, Wang T, Fu F (2015). Oral Administration of Escin Inhibits Acute Inflammation and Reduces Intestinal Mucosal Injury in Animal Models. Evid Based Complement Alternat Med.

